# Trends and Regional Differences in the Use of Maintenance Inhaled Medications in COPD: A Population-Based Study

**DOI:** 10.1007/s11606-025-09703-3

**Published:** 2025-07-16

**Authors:** Jeenat Mehareen, Yiwei Yin, Kayly Choy, Kate M. Johnson, Don D. Sin, Mohsen Sadatsafavi

**Affiliations:** 1 Respiratory Evaluation Sciences Program, Faculty of Pharmaceutical Sciences, University of British Columbia, Vancouver, BC Canada; 2 Division of Respiratory Medicine, Department of Medicine, University of British Columbia, Vancouver, BC Canada; 3 Legacy for Airway Health, Vancouver Coastal Health Research Institute, Vancouver, BC Canada; 4 Centre for Heart Lung Innovation and Division of Respirology, Department of Medicine, University of British Columbia, Vancouver, BC Canada

## INTRODUCTION

Maintenance inhaled medications, including long-acting muscarinic antagonists (LAMA), long-acting beta2-agonists (LABA), and inhaled corticosteroids (ICS), are the cornerstone of contemporary management of chronic obstructive pulmonary disease (COPD).^1^ Geographic variation in medication use for COPD has been documented at a national level, with studies suggesting that such variation may contribute to the observed variation in outcomes.^2^ However, with evolving guidelines in COPD management, our understanding of how regional variations in pharmacotherapy patterns change over time within a given healthcare system remains limited. In this study, we used population-based health data to describe temporal and geographical trends in long-acting maintenance inhaled medication use within a single healthcare system.

## METHODS

This was a retrospective cohort study in the Canadian province of British Columbia (BC). We included adults aged $\ge$ 35 with COPD based on a validated case definition^3^ and analyzed trends from 2010 to 2020. We used demographics databases and pharmacy records for all outpatient dispensed medications regardless of the payer.^4^ Within this cohort, the “index date” was the first dispensation of any (overall) or specific (each drug class) maintenance inhaled medication and marked the beginning of follow-up.

Geographic regions across BC are organized into 16 distinct health services delivery areas for planning, reporting, and implementing provincial health policies. The primary outcome was the proportion of maintenance inhaled medication users among prevalent COPD patients, overall and by medication class across those regions. Single therapies consisted of individual use of ICS, LABA, and LAMA. Combination-therapies included either single-inhalers with multiple ingredients or separate inhalers with ≥ 14 days of overlap.

Heterogeneity in medication use across regions over calendar years was visualized using boxplots. To adjust for the contribution of patient characteristics, we fitted negative binomial models (with logarithmic link function) with the total number of users as the outcome and region (dummy-coded), age, sex, urban/rural residence, and socio-economic status (SES, neighborhood income quintiles) as independent variables. We generated rate ratios (RR) and 95% confidence intervals. Heterogeneity across regions was tested using a likelihood ratio test. The study received ethics approval from Human Ethics Board at University of British Columbia (H23-00607).

## RESULTS

**Figure 1:**
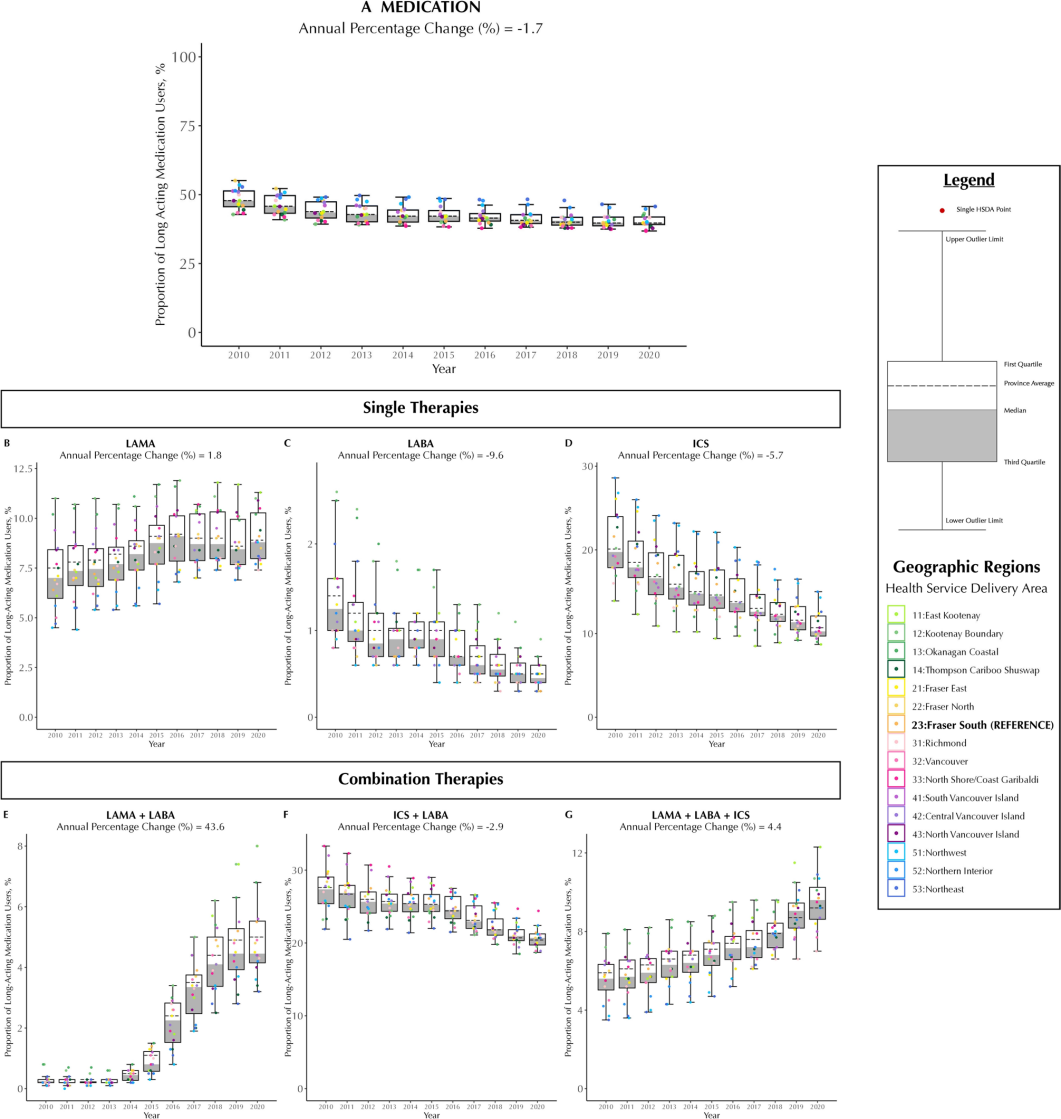
Annual proportion of maintenance inhaled medication users (**A**), further classified by single-therapies: LAMA (**B**), LABA (**C**), ICS (**D**), and combination-therapies LAMA + LABA (**E**), ICS + LABA (**F**), and LAMA + LABA + ICS (**G**) among patients with COPD in British Columbia, Canada, from 2010 to 2020, stratified by geographic region (HSDA). Abbreviations: COPD, chronic obstructive pulmonary disease; ICS, inhaled corticosteroids; LABA, long-acting beta2-agonists; LAMA, long-acting muscarinic antagonists; HSDA, Health Services Delivery Area; Q1, quartile 1; Q3, quartile 3. Note: Each dot represents an HSDA. The horizontal line cutting through the plot is the overall provincial average. The median is the line separating the upper (white) and lower (dark gray) boxes. Combination-therapies are based either on single-inhalers containing multiple ingredients or from separate inhalers with 14 days of overlap. Annual percentage change is obtained from a negative binomial regression model.

Over 11 years, the number of COPD patients using any medication increased from 77,273 to 83,157; however, the proportion of users declined from 59.1 to 41.7% (average decline 1.7%/year; Fig. 1A). Among single-inhaler therapies, ICS was used by 33.4% patients and showed the fastest decline (average 9.6%/year). Single-inhaler LABA was used by 2.3% of patients (average decline of 5.7%/year). Single-inhaler LAMA was used by 19% (increased by 1.8%/year). Among combination-therapies, 39.1% of COPD patients used ICS + LABA, followed by triple-therapy (ICS + LAMA + LABA, 14.7%) and LAMA + LABA (6.4%). The proportion of ICS + LABA users declined by 2.9%/year, whereas LAMA + LABA and triple-therapy users rose annually by 43.6% and 4.4%, respectively.

**Figure 2:**
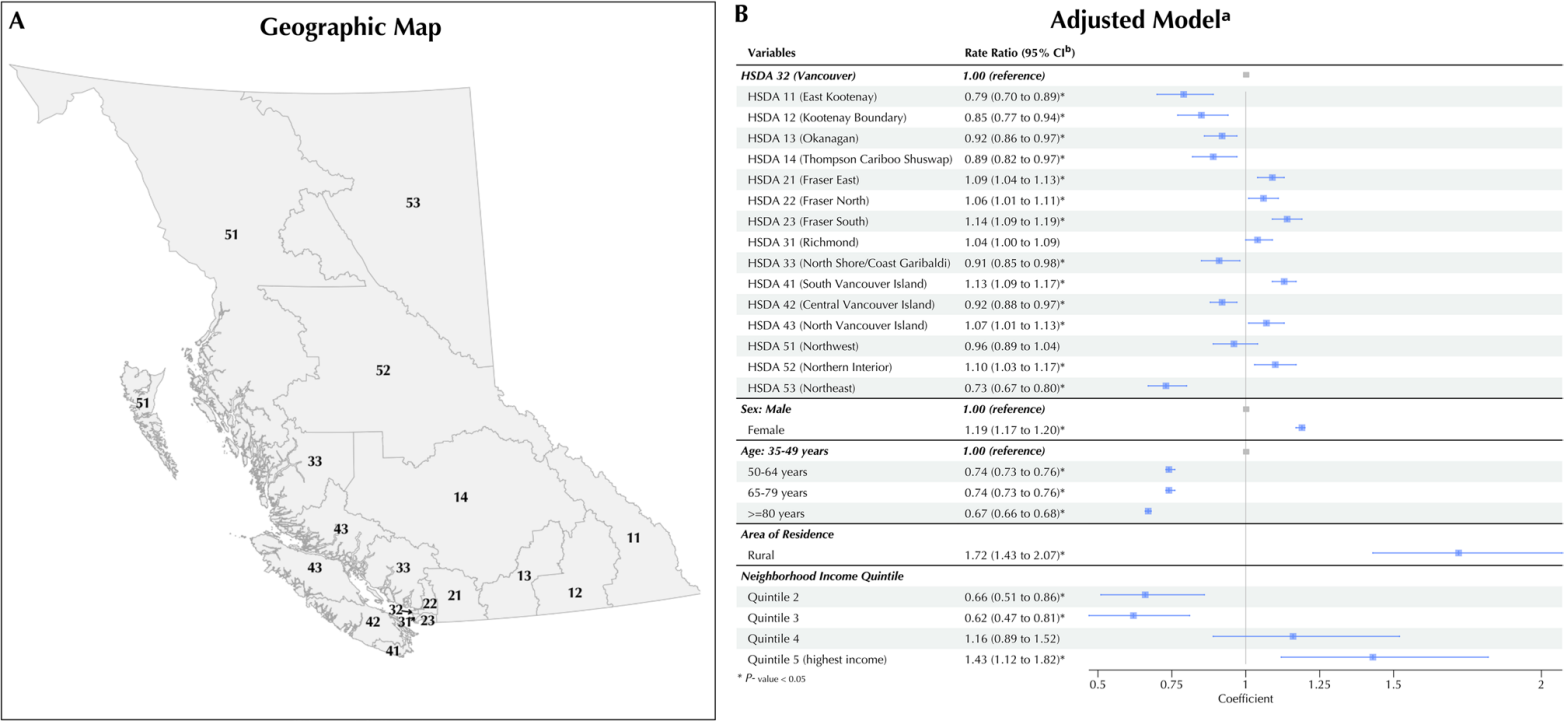
Geographic map of the regions (HSDA) (**A**) and forest plots of adjusted RRs and 95% CI of maintenance inhaled medication use (**B**). CI, confidence interval. The negative binominal regression models were adjusted for age group, sex, socio-economic status, and area of residence. Abbreviations: HSDA, Health Services Delivery Area.

Females, younger patients, and those with either lowest (compared to those in second and third quintiles) or highest (compared to the lowest category) SES were more likely to use maintenance inhaled medications. There was significant regional variability in medication use after controlling for patient characteristics (*p* < 0.01). Compared to the reference region, the RR of medication use ranged from 0.80 to 1.20 across regions (Fig. 2B).

## DISCUSSION

Our study showed an overall decline in the proportion of COPD patients using maintenance inhaled medications, primarily driven by reduced proportion of ICS and/or LABA users. However, despite the decrease, ICS-containing regimens remained the most used across all years. This highlights the potential overuse of ICS and poor prescribing practices. Meanwhile, users of LAMA-containing therapies have steadily increased, aligning with its growing recognition as the preferred treatment in COPD guidelines.^1,5^

Previous studies have shown significant regional difference in hospitalization and mortality;^6^ our reported significant regional variation in medication use may help explain part of such results. While health services are fully covered by the government, medication coverage is income-based, primarily benefitting low-income individuals.^7^ This partially explains why patients in the lowest income quintile had higher use of maintenance inhaled medications compared to those in the second and third quintiles.

The strength of this study was its full population coverage of dispensation records and a long follow-up time. However, clinical variables (i.e., symptom burden, lung function) that determine medication choice were not available. Future studies may explore how clinical and system-level factors (i.e., access to care, provider prescribing behaviors) contribute to regional and temporal differences. Investigating these factors could help inform targeted interventions for optimizing COPD management.

## Data Availability

Access to data provided by the Data Stewards is subject to approval but can be requested for research projects through the Data Stewards or their designated service providers. The following data sets were used in this study: (Vital Statistics—Deaths, Consolidation Files, PharmaNet). You can find further information regarding these data sets by visiting the PopData project webpage at: https://my.popdata.bc.ca/project_listings/23-081/collection_approval_dates. All inferences, opinions, and conclusions drawn in this publication are those of the author(s) and do not reflect the opinions or policies of the Data Steward(s).

## Declarations:

### Preprint:

Some of these findings are reported in the Supplementary Material section of a preprint (link: https://www.medrxiv.org/content/10.1101/2025.01.07.25320159v1).
